# Seroprevalence and Genotype Diversity of Hepatitis C Virus in the Caribbean—A Review

**DOI:** 10.3390/tropicalmed8070370

**Published:** 2023-07-17

**Authors:** Michelle G. Brown, John F. Lindo, Ivan E. Vickers, Kereann Nelson, Yakima Phillips, Cameil Wilson-Clarke, Samuel Gavi, Gene D. Morse, Andrew H. Talal

**Affiliations:** 1 Department of Microbiology, The University of the West Indies, Mona, Kingston 7, Jamaica; john.lindo@uwimona.edu.jm (J.F.L.); ivan.vickers02@uwimona.edu.jm (I.E.V.); kereann.nelson@uwimona.edu.jm (K.N.); yakima.phillips@uwimona.edu.jm (Y.P.); 2 Department of Basic Medical Sciences, The University of the West Indies, Mona, Kingston 7, Jamaica; cameil.wilsonclarke@uwimona.edu.jm; 3 Translational Pharmacology Research Core, Center for Integrated Global Biomedical Sciences, School of Pharmacy and Pharmaceutical Sciences, University at Buffalo, 701 Ellicott Street, Buffalo, NY 14203, USA; samuelgavi@yahoo.com (S.G.); emorse@buffalo.edu (G.D.M.); 4 Division of Gastroenterology, Hepatology, and Nutrition, Jacobs School of Medicine and Biomedical Sciences, University at Buffalo, 875 Ellicott Street, Suite 6089, Buffalo, NY 14203, USA; ahtalal@buffalo.edu

**Keywords:** seroprevalence, genotype diversity, Caribbean, HCV elimination, pan-genotype treatment, high-risk population

## Abstract

Hepatitis C (HCV) continues to present a global public health challenge, with no vaccine available for prevention. Despite the availability of direct-acting antivirals (DAAs) to cure HCV, it remains prevalent in many regions including the Caribbean. As efforts are made to eliminate HCV from the region, existing barriers, such as the high cost of DAAs and lack of an established database of HCV cases within the Caribbean, must be addressed. This review seeks to assess epidemiologic trends (seroprevalence and genotypic diversity) of HCV in the Caribbean and identify gaps in surveillance of the disease. The literature for the period 1 January 2005 to October 2022 was reviewed to gather country-specific data on HCV across the Caribbean. References were identified through indexed journals accessed through established databases using the following keywords: Caribbean, genotype distribution, and general epidemiologic characteristics. The usage pattern of HCV drugs was determined from information obtained from pharmacists across the Caribbean including Jamaica. The prevalence of HCV in the Caribbean was 1.5%; the region should therefore be considered an area of moderate HCV prevalence. The prevalence of HCV among intravenous drug users (21.9–58.8%), persons living with HIV/AIDS (0.8 to 58.5%), prisoners (32.8–64%), and men who have sex with men (MSM) (0.8–6.9%) was generally higher than in the general population (0.8–2.3%). Genotype 1 (83%) was most prevalent followed by genotypes 2 (7.2%) and 3 (2.1%), respectively. Less than 50% of countries in the Caribbean have reliable or well-curated surveillance data on HCV. Drugs currently being used for treatment of HCV infections across the Caribbean include Epclusa (sofosbuvir/velpatasvir) and Harvoni (ledipasvir/sofosbuvir). Some of these drugs are only available in the private sector and are sourced externally whenever needed. While trends point to a potentially higher prevalence of HCV, it will require well-designed random surveys to obtain better estimates of the infection seroprevalence, supported by strong public health laboratory systems. DAAs that are pan-genotypic should translate into treatments that are affordable, accessible, and available to improve cure rates and reduce the HCV burden in the population.

## 1. Introduction

According to the World Health Organization (WHO), about 1.34 million persons died from viral hepatitis in 2015 (WHO, 2017). Of these, 720,000 deaths were due to cirrhosis and 470,000 deaths were due to hepatocellular carcinoma [1]. Up to 3% of the global population, equaling approximately 170 million individuals, is estimated to be infected with chronic hepatitis C virus (HCV) [2,3]. A systematic review and meta-analysis by Salari et al. reported a global HCV prevalence of 1.8% ((95% CI, 1.4% to 2.3%) in the general population [2,3]. The goal of WHO, as stated in its HCV elimination program, is to reduce the number of new cases of HCV infection by 90% and also to achieve a decrease in worldwide deaths due to HCV from 400,000 (that occurred in 2015) to 140,000 by the year 2030 [1,4,5]. Strategies to prevent and control HCV infection include raising awareness through public education, ensuring the safety of blood transfusions, early diagnosis, and effective medical support [6].

HCV causes inflammation of the liver and was first described in 1989 [7,8]. It is a small (50 nm) enveloped virus that belongs to the Flaviridae family and Hepacivirus genus [9,10,11]. HCV has eight genotypes named 1 to 8 in order of their discovery and more than 100 subtypes defined by letters (1a, 1b, 2a, 2b, 3a, etc.) [4,9,11]. HCV genotypes are relevant to epidemiological surveillance, vaccine development, and clinical management of chronic infection [12]. Genotype 1 is the most predominant genotype circulating in the Caribbean (with subtypes 1a and 1b accounting for 70% of all cases) [4,10,11,12,13,14].

Infection with HCV may be either acute or chronic; clinically, about 60–70% of individuals infected with HCV will develop chronic disease that consists of circulating virus in the peripheral blood. HCV infects hepatocytes, which are the only known reservoir for the virus that can result in the development of inflammation and ultimately scarring (i.e., fibrosis) of the liver. Between 20 and 30 years after initial infection, 5% to 20% of individuals will develop cirrhosis and 1% to 5% of individuals will die from the consequences of cirrhosis or hepatocellular carcinoma (HCC), according to the Centers for Disease Control and Prevention [1,5,15,16].

In the Caribbean, there are several existing challenges that will thwart efforts to eliminate HCV from the region. Firstly, in non-health care settings, HCV may readily be transmitted through contaminated needles used in intravenous drug use, body piercings, and tattoos, and rarely from engaging in unprotected sexual intercourse. In the health care setting, the virus may be transmitted by the use of contaminated medical equipment, transfusion of unscreened blood/blood products, needle-stick injuries with contaminated needles, hemodialysis, and organ transplantation [17,18,19,20,21]. Therefore, existing gaps in infection prevention and control at hospitals and other health care facilities need to be identified and corrected in a timely manner in order to prevent nosocomial spread of the virus [1,4,5,6,9,11,18,19,20,21,22,23]. Additionally, existing barriers to harm reduction need to be identified and effectively addressed through, for example, increased access to sterile syringes and supplies. These interventions will likely reduce the risk of contracting HCV from injection drug use [19].

A review of the literature has revealed that limited data are available on the prevalence and genotype diversity of HCV in Caribbean countries. This may be attributed to the lack of representative sample size studies, the publication of data in non-indexed sources, or the lack of publication of data generated from studies [23,24,25]. The issues of limited data are compounded by the fact that there does not seem to be an established hepatitis virus database/registry for the region.

Unlike HBV, there is no vaccine to prevent acute and/or chronic infections with HCV, primarily because HCV mutates very rapidly, and it does not elicit protection against reinfection [26]. Drug therapy for HCV infection includes treatment with direct-acting antivirals (DAAs). DAAs are almost always universally curative, with a sustained virologic response (SVR) of about 99%. SVR is defined as undetectable HCV RNA three months after completing a course of therapy [26,27]. In addition, DAAs are taken orally for at most 12 weeks, with minimal side effects, unlike previously used HCV antivirals such as ribavirin and interferon (IFN). Pan-genotypic treatment of infections will result in a decline in HCV-related morbidity and mortality in infected patients [26,27]. WHO recommends pan-genotypic treatment of all HCV-infected individuals age ≥ 12 years old irrespective of disease stage [28]. Currently, the access price for DAAs in the Caribbean is now more affordable than before—for example, the cost of Epclusa in the US is ~USD 81,000 for a full 12-week course (drugs.com., accessed on 8 June2023), but in the Caribbean, the same drug can be accessed at a price of 1000 USD/dose (USD 3000 total for three doses). In addition, Harvoni (ledipasvir/sofosbuvir 90 mg/400 mg) is too costly and Epclusa is a better product, so it is no longer beneficial for it to be sourced. 

Taking into consideration the above background information, the primary purpose of this review is to elucidate the seroprevalence and genotype diversity of HCV in the Caribbean and to assess the public health implications for the elimination of HCV from the region.

## 2. Materials and Methods

**Figure 1:**
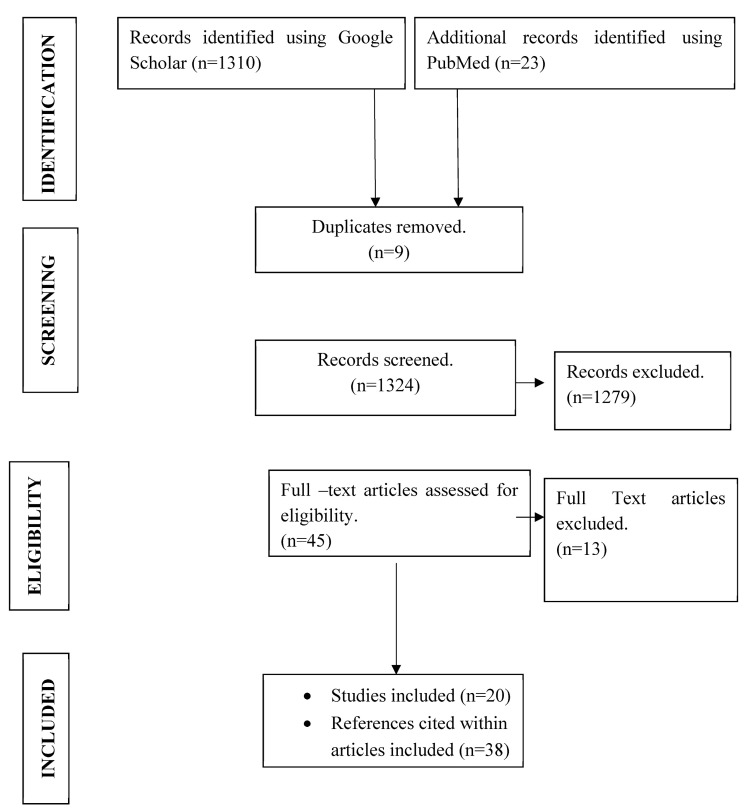
Flow diagram for study identification and selection.

We reviewed the literature without language restrictions for the period January 1, 2005 to October 31, 2022 to gather country-specific data on the prevalence and genotype distribution of HCV across the Caribbean. References were identified through indexed journals, which were found by searching PubMed and Google Scholar databases using the following terms: ‘hepatitis C/HCV genotype in the Caribbean’, ‘hepatitis C/HCV in the Caribbean’, or ‘hepatitis C genotype distribution in the Caribbean’. Overall, a total of 1333 records were identified using Google Scholar and PubMed databases, and 9 duplicate records were removed. After screening of 1324 records, 1279 were excluded because they were not relevant to the subject of HCV in the Caribbean. A total of 45 full-text articles were screened for eligibility, of which 25 unrelated articles were excluded based on their title or abstract. Therefore, we included a total of 20 articles in the review. Thirty-eight relevant references cited within the articles were also reviewed and then selected by the main author and a coauthor for inclusion in the paper (Figure 1).

Inclusion Criteria: Articles published before 1 January 2005, except for Jamaica due to the availability of limited data for the prevalence of HCV in selected populations. 

Exclusion Criteria: Articles published after 31 October 2022. Articles unrelated to the seroprevalence or genotype diversity of HCV in the Caribbean were excluded. Duplicate records were removed.

It should be noted that an extended search of the literature prior to 2005 was performed specifically for Jamaica, to include available data.

Information relating to the prescription of DAAs was obtained from pharmacists across the Caribbean. These pharmacists communicated with each other under the umbrella of the Caribbean Association of Pharmacists (CAP). A formal request was made to the organization for a mini survey to be conducted on HCV. The following questions were asked:

1. What country do you practice in?
2. What treatments are used for HCV in your country?

This mini survey was sent to the 194 members of the CAP; however, only 26 pharmacists responded using ‘WhatsApp Messenger’ or email. Of this number, information was only received for 14 countries. Information on the utilization pattern of DAAs in the Caribbean was obtained from distribution companies via telephone conversation, ‘WhatsApp Messenger’, or email.

## 3. Results

### 3.1. Regional Prevalence of HCV

A search of the literature revealed that across the Caribbean, published data on HCV were available from Cuba, Jamaica, Haiti, the Dominican Republic, Puerto Rico (Greater Antilles), Guadeloupe, and Martinique (Lesser Antilles). The overall prevalence of 1.5% (with a viraemic rate of 70%) in the general population has been reported for HCV in the Caribbean, based on a systematic review of the literature by Petruzziello et al. Countries included in this analysis included Cuba, the Dominican Republic, and Puerto Rico. This prevalence was calculated from the sum of data reported from the region divided by the number of countries within the region. Prevalence of HCV rates ranging between 0.55% and 6.3% have also been reported in the literature [29,30,31,32].

Overall, genotype 1 was found to be the most predominant genotype (83%) in circulation in the Caribbean, followed by genotypes 2 (7.2%), 3 (2.1%), 4 (0.6%), 6 (0.1%), and 5 (nil), respectively [4,10,11,12,13,14,24,32,33] (Table 1).

### 3.2. Prevalence of HCV

#### 3.2.1. Greater Antilles

Seroprevalence among Latino veterans in Puerto Rico was 2.3%; data were obtained from a retrospective review of 65, 684 records obtained from the Veteran Affairs Health Care System (VAHCS) for the period 1 January 2002–31 December 2009 [32,33,34].

A probability cluster design study reported a prevalence of 6.3% (95% CI: 3.6–10.9%) among 964 household representatives aged 21–64 years in San Juan [29]. In 2016, the prevalence of HCV in Puerto Rico was reported as 2.3% in the general population based on a systematic review of the literature for the period 2000–2015 by Petruzziello et al. [32].

**Figure 2:**
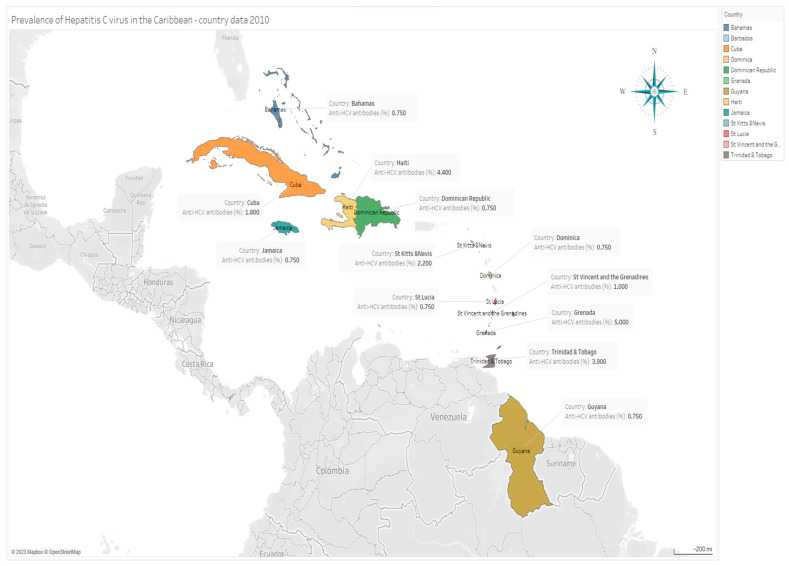
Prevalence of HCV in the Caribbean in 2010, by country.

In Cuba, HCV prevalence rates of 0.8% and 1.8% were reported based on data from country reports and population prevalence from population-based studies in 2010 and 2016, respectively [32,33,34]. In Haiti and Jamaica, HCV prevalence was reported to be 4.4% and 0.75%, respectively, in the general population [30]. In Jamaica, for example, 20,250 HCV cases were reported, and with a population of 2.7 million inhabitants, HCV prevalence was estimated at 0.75%. Similarly, based on the population of 10,500 HCV-infected individuals in the Dominican Republic in 2014, the prevalence of HCV was estimated at 1.0% (0.8–2.4%) [35] (Table 2 and Figure 2).

#### 3.2.2. Lesser Antilles

The reported prevalence of anti-HCV antibodies was highest in Grenada (5.0%), followed by Trinidad and Tobago (3.9%), St Kitts and Nevis (2.2%), and St Vincent and the Grenadines (1.0%). Barbados, Bahamas, Dominica, and Guyana had a reported HCV prevalence of 0.75% [30] (Table 2).

#### 3.2.3. Prevalence of Hepatitis C Virus in the Greater and Lesser Antilles

Overall, prevalence varied from 0.75% to 5.0%. In the Greater Antilles, the highest prevalence was found in Cuba (1.8%), while both in Jamaica and the Dominican Republic, the prevalence was <1.0%. Similarly, in the Lesser Antilles, the highest prevalence was found in Grenada (5.0), while for the other countries, prevalence varied from 0.75% to 3.9%.

### 3.3. Treatment of HCV

DAAs currently being used for treatment of HCV infections across the Caribbean include Epclusa (sofosbuvir/velpatasvir) and Harvoni (ledipasvir/sofosbuvir). Some of these medications are only available in the private sector and are sourced externally whenever needed (Table 3). The lack of epidemiological studies has impeded elimination efforts. The Caribbean Public Health Agency (CARPHA), in an effort to eliminate HCV from the region, has recommended the following: enhancement or development of national hepatitis strategic plans; increased diagnostic testing; bridging the gap in infection control and control capacity in health care facilities; and a focus on barriers to harm reduction [19]. In the Dominican Republic, efforts to eliminate HCV have included an enhancement of screening activities, such that screening is conducted for employment applications, prior to medical procedures and marriages, and for pregnant females, rather than only for blood banking [36]. In Puerto Rico, discriminatory restriction on access to HCV treatment that was affecting more than 1 million Puerto Ricans covered by the Medicaid program has been lifted [37].

Another limitation to HCV elimination in the Caribbean is the lack of a registry of the drug distribution or prescription for HCV medications in the region. However, we collected information directly from the pharmaceutical suppliers that market the relevant drugs. Unfortunately, however, the data are not standardized.

#### 3.3.1. Greater Antilles

No information relating to DAAs for HCV was obtained for Cuba, Puerto Rico, Haiti, and the Dominican Republic. In Jamaica, older therapies included Pegasys (peginterferon alpha-2a) and ribavirin; these have been replaced by newer therapies—Epclusa (sofosbuvir/velpatasvir 400/100) and Harvoni (ledipasvir/sofosbuvir 90/400).

#### 3.3.2. Lesser Antilles

For the protocol members of the Organisation of Eastern Caribbean States (OECS) (Antigua and Barbuda, Dominica, Grenada, St Kitts and Nevis, St Lucia, and St Vincent and the Grenadines), DAAs are ordered from the USA as needed. Similarly, for associate members of OECS (Anguilla, Guadeloupe, Martinique, and the British Virgin Islands), DAAs are ordered from the USA whenever needed. For the years 2020 to 2022, only one case of HCV infection was identified and treated with Epclusa (sofosbuvir/velpatasvir 400/100) in any full or associate OECS country.

In the Bahamas, private sector patients are treated with Epclusa (sofosbuvir/velpatasvir 400/100) and Harvoni (ledipasvir/sofosbuvir 90/400). If treatment for HCV infection is required in Barbados, Epclusa (sofosbuvir/velpatasvir 400/100) is supplied.

### 3.4. Prevalence of HCV in Special Populations

Persons considered to be ‘at risk’ for infection with HCV include intravenous drug users, prisoners, and men who have sex with men. All these groups may feel stigmatized and hesitant to seek medical treatment and diagnostic testing [19]. In addition, the majority of seropositive persons may be unaware of their infection status and may not seek medical attention until they become seriously ill, oftentimes resulting from liver decompensation, resulting in increased morbidity and mortality from the infection. Therefore, published prevalence rates in these groups are at best underestimates. Public/national education on the risk factors, modes of transmission, clinical presentation, and methods of control and prevention of infection with HCV is needed [20,21,22,23].

A review of the literature by Grebely in 2019 found that in the Bahamas, Bermuda, the Dominican Republic, Haiti, and Jamaica, there was evidence of injection drug use but no estimate of the prevalence of HCV infection in this high-risk group, so it is not known how common this mode of transmission is. However, there were no reports of injection drug use in several countries including Antigua and Barbuda, Barbados, Cuba, Dominica, Grenada, Saint Kitts and Nevis, Saint Lucia, St Vincent and the Grenadines, and Trinidad and Tobago [38].

#### 3.4.1. Greater Antilles

In Puerto Rico, HCV prevalence was significantly higher than in the general population for study participants with a history of heroin use (39.2%), cocaine use (39.6%), and imprisonment (32.8%) [29]. In metropolitan San Juan, varying prevalence (78.4–89.0%) of HCV antibodies was reported among injection drug users; the prevalence is slightly lower in the rural population (78.4%) [39,40,41,42]. This prevalence was more than two times higher than that reported in southern Puerto Rico by Colon-Ruiz et al. in a cross-sectional data analysis of medical records of HCV-infected adults for the period January 2005–March 2013 [43].

Overall, in the Caribbean—except for Puerto Rico, with a reported prevalence of 89%—intravenous drug abuse (IVDA) is not a major public health problem. In Puerto Rico, 17% of IVDA individuals are positive for HIV, with 95% co-infected with HCV. This may be attributed to increased injection and needle sharing and decreased sterile syringe programs [20].

In Jamaica, a prevalence of HCV antibodies in NIDU (non-injection drug use) individuals of 1.7% was reported among residents of a detoxification unit by Smikle et al. [44].

Further, the prevalence of HCV among hemophiliacs in Jamaica was reported to be 41%, and associated risk factors included increasing age, disease severity, and frequency of blood transfusions [45].

HCV infection was identified as a public health problem in Cuba in the 1990s. Despite universal blood donor screening, which was achieved in 1995, the infection is still found in multitransfused patients [46]. In 2005, the prevalence of HCV antibodies among 318 Cuban patients who were transfused with ≥10 units of allogeneic blood/blood products on ≥ 2 occasions was reported to be 51.6% [46]. A systematic review of the literature (2000–2013) by Alonso et al. cited only four studies from the Dominican Republic, where the prevalence of HCV among gay and transsexual individuals and men who have sex with men ranged from 0.8% to 6.8%, and two studies on injection drug use in Puerto Rico reported an HCV prevalence of 39% and 89%, respectively [23,47,48].

For the period 2005–2013, in the southern area of Puerto Rico, the prevalence of anti-HCV antibodies among adults that received blood transfusions was found to be 33% [43].

#### 3.4.2. Lesser Antilles

In Guadeloupe, among a general clinic-based population, a low prevalence of HCV of 0.55% was found, in comparison to a prevalence of 0.8% found previously among blood donors; risk factors for HCV acquisition included gynecological surgery, endoscopy, tattooing, shaving, intravenous drug use (IVDU), and familial exposure [31].

## 4. Discussion

Overall, limited data currently exist on the prevalence and genotype distribution of HCV in the Caribbean. This limitation has been highlighted by several systematic reviews [25,32,39,40,49]. The epidemiological burden of HCV in the region needs to be fully assessed as only a few estimates are available [50]. The data deficiency may be attributed to the lack of representative study sample sizes, the publication of data in non-indexed sources, or the lack of publication of data generated from studies [23,24,32]. Updated data are needed to reflect the true prevalence and assessment of the burden of HCV infection in the general and ‘high-risk’ populations in the region.

The estimated prevalence of HCV in the Caribbean was 1.5%, with genotype 1 being the most dominant genotype (83%). Similar prevalence (1.5–3.5%) has been seen in other regions considered to have moderate prevalence of HCV, including East, South, and Southeast Asia; West and East Africa; North Africa; the Middle East; Southern and Tropical Latin America; Australasia; and Eastern Europe [4,29,30,31,32]. In consideration of these data, the Caribbean region should therefore be considered an area of moderate HCV prevalence. Of note, the prevalence of HCV in the Caribbean is 0.3% less than the global prevalence of 1.8% and 5.6% less than that reported from the continent of Africa, which is reported to have the highest prevalence of HCV [3].

The persistence of HCV may be attributed to the practices of ‘at-risk’ or vulnerable populations including illicit drug users (injection and non-injection), men who have sex with men, sex workers, and prison inmates. A relatively high disease burden of HCV has been reported in these key populations [23,47,48].

Despite the fact that no data were found on drug use in several Caribbean countries except for Puerto Rico, there is reported evidence of injection drug use, but no estimate of the prevalence of HCV infection in this high-risk group, in the Bahamas, Bermuda, the Dominican Republic, Haiti, and Jamaica [38]. Additionally, injection drug use is uncommon in most Caribbean countries [49,50]. Based on the high prevalence of HCV in injection drug users in Puerto Rico, needle exchange and drug treatment programs are indicated as a method to reduce transmission of HCV.

Blood transfusions that occurred prior to the introduction of widespread screening of blood/blood products in the mid-1990s may be considered a risk factor in persons already infected. This may lead to persistence of the infection in multiple-transfused persons, such as hemophiliacs.

Across the Caribbean, drugs currently being used for treatment of HCV infections include Epclusa (sofosbuvir/velpatasvir 400/100) and Harvoni (ledipasvir/sofosbuvir 90/400). In some countries, such as St Lucia and the Virgin Islands, there are no reported active cases of HCV. The COVID-19 pandemic has adversely impacted routine national health services in individual countries, resulting in limited routine laboratory testing and treatment for HCV. A high mortality rate has been found in patients co-infected with HCV and COVID-19 and with cirrhosis, as they all contribute to a decline in liver function [6]. As such, treatment of HCV infection is important to remove an additional burden from COVID-19 patients.

There exists a need for the establishment of an effective epidemiological surveillance system to provide information needed for planning, implementation, and intervention strategies for HCV infection. Furthermore, in some populations within the Caribbean, such as drug users, access to medical and other existing therapy is limited [20,21,22,23]. Therefore, there is a need to enhance or develop HCV prevention plans that clearly outline actions necessary to control and ultimately eliminate the virus.

Data on the epidemiology of HCV across the region can provide crucial information to policymakers and public health agencies, such as CARPHA, on the need for targeted interventions for prevention and treatment. Studies to assess the scope of chronic hepatitis, the burden of disease, and seroprevalence, in both the general and most at-risk populations, are necessary. Countries in the English-speaking Caribbean are all members of the Caribbean Community (CARICOM), and they often implement common health care policies. Continuous surveillance and intensive prospective epidemiologic research studies should be performed to inform policies aimed at the elimination of this growing hepatitis epidemic.

The establishment of a viral hepatitis C database/registry in the region is highly recommended. Based on the 2021 WHO Technical Report for the elimination of HCV (and other types of viral hepatitis) as a public health problem, goals that should be realized for country validation include the following: annual incidence of ≤5 cases/100,000 inhabitants; and ≤2 cases/100 persons who inject drugs (PWID). Further, with respect to testing and treatment, the goal is the diagnosis of ≥90% of persons and the treatment of ≥80% of those diagnosed with HCV. Prevention strategies include 0% unsafe injections, 100% blood safety, and 300 needles/syringes/PWID/year [51].

An excellent mechanism to eliminate hepatitis C is through CARICOM, where CARPHA establishes health interventions and research policies for all 15 full and 5 associate member countries. However, the first step is the elucidation of the epidemiology of the infection in individual countries based on rigorous prospective studies to include high-risk populations.

In low-and middle-income countries with limited infrastructure, such as India, Cambodia, and Indonesia, recent efforts to eliminate HCV infection included political support and decentralized programs. These approaches have enabled intensive low-cost screening and a reduction in the cost of medication and have resulted in a cure rate exceeding 90% in 120 individuals [52].

In European countries, such as France, plans to eliminate HCV infection include the availability of oral antivirals to members of various ‘high-risk ‘populations such as persons living with HIV and men who have sex with men, for the years 2015 and 2016, respectively. Universal HCV treatment, with full coverage by French National Health Care, was introduced in 2017. This intervention has resulted in the treatment of about 60,000 patients during the period 2017 to 2022 [53].

### 4.1. Limitations

There is a lack of published papers on the prevalence or genetic diversity of HCV in the pediatric population in the Caribbean. This lack of data further highlights the need for studies on HCV in the pediatric population in the Caribbean, which will help to guide national policies to eliminate the virus. In addition, in the Caribbean, the percentage of the general detained population with HCV antibodies was unknown since no sources of data were identified for the region. Individuals incarcerated in prisons usually have a known history of drug use (injecting or non-injecting), promiscuity, homosexuality, and tattooing, all of which increase the risk of HCV transmission. Current, well-designed studies are needed to ascertain the true prevalence of HCV in these populations in the region in an effort to completely eliminate HCV from the region.

The variation in prevalence estimates among populations maybe attributed to the variation of the selection criteria used in the various published studies and systematic reviews. This includes sample size and availability or lack of data.

### 4.2. Future Directions

Increased diagnostic capacity, including the introduction of molecular tests to diagnose and confirm HCV infection, is necessary. For example, screening for HCV in Jamaica is conducted by serology, and samples that test positive for HCV antibodies are shipped to diagnostic laboratories overseas for confirmatory testing, at a cost to the patient. Fortunately, the access price of DAAs in the Caribbean is now more affordable than before—for example, the cost of Epclusa in the US is ~USD 81,000 for a full 12-week course (drugs.com), but in the Caribbean, the same drug can be accessed at a price of 1000 USD/dose (USD 3000 total for three doses). In addition, Harvoni (ledipasvir/sofosbuvir 90 mg/400 mg) is too costly, and Epclusa is a better product, so it is no longer beneficial for Harvoni to be sourced. 

In addition, there is a need for the widespread use of rapid, low-cost diagnostic tests; these should be introduced as screening tools in hospitals, health centers, and point-of-care sites.

The introduction of DAAs for HCV, coupled with pan-genotypic treatment of HCV infection, has resulted in effective management of patients [54]. Therefore, in the Caribbean, the therapeutic focus for HCV can now be on complete elimination of the virus from the region. Conducting seroprevalence and molecular studies specifically targeting the Caribbean would have great benefit in identifying and improving public health management of hepatitis infections in the region.

## Figures and Tables

**Table 1:** Genotype distribution of HCV in 3 Caribbean countries based on published studies.

Country	Study Population	Predominant Genotype(s) (%)	Sample Size	Reference
Cuba	--	G1 (98.0)	--	[32]
Puerto Rico	--	G1 (82.1)	--	[32]
Dominican Republic	--	G1 (62.6)	--	[32]
French Island Guadeloupe	General clinic-based	G1 (80.0) G2 (20)	2200	[31]

Genotype 1 was most prevalent in studies cited; -- indicates that information was not present in the literature.

**Table 2:** Prevalence of hepatitis C virus in the Caribbean in 2010, by country *.

Country	Location	Anti-HCV Antibodies (%)	Absolute No. Infected **
Grenada	Lesser Antilles	5.0	5150
Haiti	Lesser Antilles	4.4	448,272
Trinidad and Tobago	Lesser Antilles	3.9	50,583
St Kitts and Nevis	Lesser Antilles	2.2	1232
Cuba	Greater Antilles	1.8	202,842
St Vincent and the Grenadines	Lesser Antilles	1.0	1180
Bahamas	Lesser Antilles	0.75	2250
Barbados	Lesser Antilles	0.75	2100
Dominica	Lesser Antilles	0.75	593
Dominican Republic	Greater Antilles	0.75	66,713
Guyana	Lesser Antilles	0.75	5633
Jamaica	Greater Antilles	0.75	20,250
St Lucia	Lesser Antilles	0.75	1232

* The available prevalence is shown for respective Caribbean countries based on published data adapted from Lavenchy 2011. The seroprevalence of HCV antibodies across the region varied from 0.75% to 5.0%. ** Indicates absolute number of persons that were found to be seropositive for HCV antibodies.

**Table 3:** Information relating to use of HCV drugs across Caribbean islands.

List of Caribbean Countries	Comments
Antigua and Barbuda	-----------
Aruba	Routine use of Epclusa (sofosbuvir/velpatasvir 400/100)
Bahamas	- In the private sector, patients are treated with Epclusa (sofosbuvir/velpatasvir 400/100) and Harvoni (ledipasvir/sofosbuvir 90/400)
Barbados	- Epclusa (sofosbuvir/velpatasvir 400/100) is supplied if needed for treatment of HCV-infected patient (s)
Bermuda	Harvoni (ledipasvir/sofosbuvir 90/400)
Curacao	Routine use of Epclusa (sofosbuvir/velpatasvir 400/100)
Virgin Islands	Currently no cases of hepatitis C
Guyana	Discontinued use of Epclusa (sofosbuvir/velpatasvir 400/100) and current use of cheaper combination (daclatasvir/velpatasvir)
Jamaica	- Use of Epclusa (sofosbuvir/velpatasvir 400/100) and Harvoni (ledipasvir/sofosbuvir 90/400) for those with age more than 6 years—ordered from USA/through local distributor
Saint Lucia	Currently no cases of hepatitis C on the island
Saint Maarten	Routine use of Epclusa (sofosbuvir/velpatasvir 400/100)
The protocol members of the Organisation of Eastern Caribbean States (OECS) are Antigua and Barbuda Commonwealth of Dominica Grenada Montserrat St. Kitts and Nevis Saint Lucia St. Vincent and the Grenadinesines. The associate members of the OECS are: The British Virgin Islands Anguilla Martinique Guadeloupe.	One patient for the period 2020–2022 received Epclusa (sofosbuvir/velpatasvir 400/100)

There are limited data available on treatment for HCV in the Caribbean. A dashed line indicates that no information is available for that country. Data were collected via a mini survey from members of the Caribbean Association of Pharmacists (CAP). Members responded via email or ‘WhatsApp’. Abbreviations: HCV, hepatitis C.

## Data Availability

Data may be accessed at the following websites: http://www.pubmed.gov (accessed on 20 June 2022); http://www.googlescholar.com (accessed on 30 June 2022; http://www.drugs.com (accessed on 8 June 2023).
